# Tumor Necrosis Factor-Alpha Targeting Can Protect against Arthritis with Low Sensitization to Infection

**DOI:** 10.3389/fimmu.2017.01533

**Published:** 2017-11-14

**Authors:** Nadia Belmellat, Luca Semerano, Noria Segueni, Diane Damotte, Patrice Decker, Bernhard Ryffel, Valérie Quesniaux, Marie-Christophe Boissier, Eric Assier

**Affiliations:** ^1^UMR 1125 INSERM, Bobigny, France; ^2^Sorbonne Paris Cité Université Paris 13, Bobigny, France; ^3^Service de Rhumatologie, Groupe Hospitalier Avicenne—Jean Verdier—René Muret, APHP, Bobigny, France; ^4^INEM UMR7355, CNRS, University of Orléans, Orléans, France; ^5^Service de pathologie Hôpitaux Universitaires Paris Centre, APHP, Université Paris Descartes, Paris, France; ^6^IDM, University of Cape Town, Cape Town, South Africa

**Keywords:** tumor necrosis factor, vaccine, rheumatoid arthritis, infection, host-defense

## Abstract

Tumor necrosis factor-alpha (TNF-α) blockade is an effective treatment for rheumatoid arthritis (RA) and other inflammatory diseases, but in patients, it is associated with reduced resistance to the infectious agents *Mycobacterium tuberculosis* and *Listeria monocytogenes*, among others. Our goal was to model infection and arthritis in mice and to compare etanercept, a currently used anti-TNF-α inhibitor, to an anti-TNF-α vaccine. We developed a murine surrogate of the TNF-α kinoid and produced an anti-murine TNF-α vaccine (TNFKi) composed of keyhole limpet hemocyanin conjugated to TNF-α, which resulted in anti-TNF-α antibody production in mice. We also used etanercept (a soluble receptor of TNF commonly used to treat RA) as a control of TNF neutralization. In a mouse model of collagen-induced arthritis, TNFKi protected against inflammation similar to etanercept. In a mouse model of acute *L. monocytogenes* infection, all TNFKi-treated mice showed cleared bacterial infection and survived, whereas etanercept-treated mice showed large liver granulomas and quickly died. Moreover, TNFKi mice infected with the virulent H37Rv *M. tuberculosis* showed resistance to infection, in contrast with etanercept-treated mice or controls. Depending on the TNF-α blockade strategy, treating arthritis with a TNF-α inhibitor could result in a different profile of infection suceptibility. Our TNFKi vaccine allowed for a better remaining host defense than did etanercept.

## Introduction

Tumor necrosis factor-α (TNF-α) is a central mediator of inflammation, tumor growth control, autoimmunity, and immune response to infection (1). In rheumatoid arthritis (RA) and spondyloarthritis, two frequent and severe diseases (2), TNF-α mediates a wide variety of effector functions, including the production of pro-inflammatory cytokines and chemokines, activation of immune cells and angiogenesis.

Two types of TNF-α antagonists are used for treating rheumatic diseases, including RA and spondyloarthritides: the soluble TNF-α receptor 2 etanercept and monoclonal antibodies such as infliximab, adalimumab, golimumab, or pegylated-IgG-Fc fragments such as certolizumab. Anti-TNF-α drugs have greatly changed the treatment of RA in terms of clinical control and articular damage prevention. However, these treatments have limitations, such as high cost, frequent therapeutic failures (only 20% of RA patients achieve sustained remission) (3), and increased risk of serious infections, such as reactivation of pulmonary and extrapulmonary tuberculosis (4, 5).

Recently, we developed an alternative strategy of TNF neutralization by a vaccination approach. A human TNF-α vaccine (hTNF-K) was obtained by chemically coupling the human cytokine to the carrier protein keyhole limpet hemocyanin (KLH) (6). Preclinical studies showed that vaccination with hTNF-K reduced arthritis development in a human TNF-α transgenic mouse (TTG) model, with a dose effect depending on level of anti-TNF-α antibody production (7–9). Anti-TNF-α antibody production was reversible and could not be stimulated by TNF-α administration, because this approach did not induce any cellular-mediated immunity against TNF-α (10). A first clinical trial was conducted with RA patients resistant to TNF antagonists and showed promising results (11).

In previous studies, we demonstrated that anti-human TNF-α vaccination activates resting autoreactive B cells to produce anti-TNF-α antibodies. The carrier protein (KLH) possesses the T epitopes able to activate T cells, thereby providing the necessary help for anti-TNF-α antibody production. No T cell being directly sensitized to TNF-α has two major consequences. First the antibody production is self-limited, with serum antibody titers showing a bell-shaped curve, with peak concentration around day 50 in mice and between day 112 and 140 in humans. Second, any increase in self-produced TNF-α (e.g., during infection) does not elicit any anti-TNF-α antibody response in immunized subjects. However, the risk of infection with the hTNF-K treatment could not be easily studied in the TTG mice because antihuman TNF-α antibodies are usually species-specific and do not cross-react with murine TNF-α.

To evaluate the risk of infection using anti-TNF-α strategies, we compared a vaccine directed against murine TNF-α we developed by coupling murine TNF-α to KLH (TNFKi) and etanercept, the anti-TNF-α treatment associated with the lowest occurrence of infections, namely iatrogenic tuberculosis (12, 13). Our data reveal the protective effect of TNFKi in two mouse models of arthritis. Furthermore, we show that, in contrast to etanercept, TNFKi had limited effects on the host resistance to *Listeria monocytogenes* and *Mycobacterium tuberculosis* infection, as reflected by a differential local immune response to the pathogens. Hence, depending on the anti-TNF-α inhibition strategy, TNF-α inhibition could control chronic inflammation without modifying the pathogen-induced immune response and, therefore, have no deleterious effect.

## Materials and Methods

### Vaccine Design

Murine TNF-α was from PeproTech (315-01A-1000UG, PeproTech France). KLH (77600, Thermo Scientific Pierce), GMBS (N-[g-maleimidobutyryloxy]), succinimide ester (22309 Pierce), and SATA [*N*-succinimidyl *S*-acetylthioacetate (26102 Pierce)] were from Thermo Scientific Pierce, France. TNFKi resulted from the chemical coupling of murine TNF-α to the carrier protein KLH, by using heterobifunctional cross-linkers that react with primary amines. TNFKi was prepared as follows: 4 mg murine TNF-α was derivatized with 1.2 mg SATA for 2 h at room temperature. In parallel, 20 mg KLH was derivatized with 1.76 mg GMBS for 30 min. Cross-linker quantities were calculated to modify half of the lysine residues. For coupling, 1 mol KLH-GMBS was incubated with 20 mol TNF-SATA for 2 h at room temperature in the presence of 5 mM hydroxylamide (23225 Thermo Scientific Pierce). The heterocomplex (TNF-KLH) was dialyzed overnight against phosphate-buffered saline (PBS) by use of a Microcon tube (YM100 Millipore). TNFKi was sterilized by centrifugation in an Ultrafree-CL 0.22-µm tube (FC40GV 0S Millipore), then stored at 4°C. Three batches were prepared successively for different experiments. They were validated biochemically by ELISA and western blot analysis. Batches were systematically tested *in vivo* in the collagen-induced arthritis (CIA) model.

### Mice

For CIA, 36 DBA/1 male mice (6 weeks old) were used, and for collagen antibody-induced arthritis (CAIA), 24 C57Bl/6 male mice (6 weeks old) were used, all purchased from Janvier Laboratory (France). Mice were randomly assigned to treatment groups and randomly distributed to cages. Body weight was monitored weekly. *L. monocytogenes* (LM) infection experiments involved 40 C57Bl/6 female mice (8 weeks old, Janvier Laboratory) and 10 TNF-α^−/−^ mice (TNF^−/−^) (14) backcrossed at least 8–10 times on a C57BL/6 genetic background, bred, and housed in the Transgenose Institute animal facility (CNRS UPS44, Orleans, France). *M. tuberculosis* (Mt) infection experiments involved 60 C57Bl/6 female mice (8 weeks old, Janvier Laboratory) and 7 TNF^−/−^ mice. Mice were kept in isolators in a biohazard animal unit. Infected mice were monitored every day for clinical status and weighed twice weekly. Mice were randomly assigned to treatment groups and randomly distributed to cages (except for naïve uninfected mice).

### Arthritis Models

DBA/1 mice used for CIA were injected with a 1:1 emulsion of bovine type II collagen (CIIb, 50 μg/mice) and complete Freund’s adjuvant (Difco, France) on day 0 (total volume injected in the tail: 100 µl). A boost was given on day 21 with 100 µl of a 1:1 emulsion of CIIb (50 μg/mice) and incomplete Freund’s adjuvant (IFA, Difco, France). Arthritis onset occurs at about 30–35 days after the first collagen injection (15).

C57Bl/6 mice used for CAIA were intraperitoneally injected with 5 mg of a cocktail of five monoclonal anti-collagen antibodies on day 0 (53100 Arthrogen-CIA Arthritogenic Monoclonal Antibody, Gentaur, Belgium) and lipopolysaccharide (50 µg, *E. coli* 0111:B4 strain) on day 3. Arthritis onset occurs at about 3–4 days after anti-collagen antibody injection and peaks at about day 7–10 (16).

Clinical signs of arthritis were evaluated with blinding to treatment three times per week. Arthritis was monitored in all four paws. For each mouse, the clinical severity of arthritis was scored as 0, normal; 1, erythema; 2, swelling; 3, deformity; and 4, ankylosis in 10 joints or groups of joints: three joints of the two hind limbs (toes, tarsus, ankle) and two joints of the two forelimbs (digits, wrist). The maximum score for each of the 10 joints was 4, so the maximum individual arthritis clinical score on a given day was 40 (17). The mean arthritic score on each day of clinical observation was calculated for each treatment group.

For histology, both left hind limbs of each mouse were collected, fixed, decalcified, dehydrated, and transferred to paraffin blocks. Slides of 7 µm thickness were stained with hematoxylin and eosin before optical microscopy observation. At least 10 fields per section were evaluated with blinding to treatment. In each joint, two variables were separately assessed on a 4-point scale (0–3, 0 indicating a normal joint, and 3, maximally severe arthritis). The first variable was inflammation, as reflected by synovial membrane thickness (synovial proliferation) and inflammatory cell infiltration. Inflammation was scored as 0, no synovial proliferation and no inflammatory cell infiltration; 1, limited or absent synovial proliferation with inflammatory cells infiltrating ≤5% of synovial membrane; 2, synovial proliferation with inflammatory cells infiltrating between 5 and 50% of synovial membrane; and 3, massive synovial proliferation with inflammatory cells infiltrating >50% of synovial membrane. The second variable was joint destruction (bone erosions, cartilage thickness, and cartilage unevenness). Joint destruction was quantified as 0, no bone erosion, smooth cartilage surface with conserved thickness; 1, presence of cartilage erosion or unevenness or thinning involving ≤50% of cartilage surface with absent or single bone erosion; 2, multiple cartilage erosions or cartilage thinning involving >50% of cartilage surface and/or >1 bone erosions involving ≤50% of articular surface; and 3, complete derangement of articular structure. For determining prevalence, histological inflammation or destruction was defined as inflammation or destruction score ≥0.5 (17).

For clinical and histological evaluation of arthritis, blinding was ensured by a random distribution of treatments inside cages, limited by the need to have each treatment group represented, and by the use of tables including only cages and mouse numbers.

### Anti-TNF Immunization in CIA Model

Immunization involved the anti-mouse TNF-α vaccine (TNFKi) emulsified in IFA. DBA/1 mice (*n* = 6) were vaccinated intramuscularly with different doses of TNFKi (20, 10, or 5 µg) on days −21, −7, and 7. In the same experiment, one group of mice (*n* = 6) were intraperitoneally injected three times/week with 30 mg/kg etanercept (PAA0252P1, Pfizer) from day 22. Negative controls were mice receiving intraperitoneal injections of PBS or intramuscular injections of KLH (10 µg) emulsified in IFA. KLH and PBS were administered on the same time schedule as TNFKi or etanercept treatment, respectively.

### Infection Protocols

*Listeria monocytogenes* (L028) was grown in BHI medium (Brain Heart Infusion, Difco Laboratories). Aliquots of *M. tuberculosis* H37Rv kept frozen at −80°C were thawed, diluted in sterile saline containing 0.05% Tween 20, and clumping was disrupted by 30 repeated aspirations through a 26-G needle (Omnican, Braun, Germany). Pulmonary infection involved delivering 2250 colony formation units (CFU) of *M. tuberculosis* H37Rv/lung into nasal cavities of mice under xylazine–ketamine anesthesia, as verified by determining bacterial load in the lungs on day 1 postinfection.

Mice were vaccinated before infection with four TNFKi intramuscular injections (10 µg) on days −44, −31, −17, and −4. One group of mice (*n* = 10) received intraperitoneal injections of etanercept (30 mg/kg) twice a week from days −4 to day 52 before and during infection. Controls were untreated (naïve) mice, vaccinated with KLH, or received intraperitoneal injections of PBS. All groups comprised 10 mice per group except for TNF^−/−^ mice (*n* = 7). On day 25 postinfection for all TNF^−/−^ mice or on day 28 for other groups, 5 animals were killed; lung, livers, and spleens were harvested; and the number of viable bacteria in organ homogenates was determined by plating serial dilutions in duplicate onto Middlebrook 7H11 (Difco) agar plates containing 10% OADC and incubating at 37°C. Colonies were counted at 3 weeks and results are expressed as log_10_ CFU per organ. Similar analyses were performed on day 56 (established infection) with the remaining mice.

A similar vaccination schedule was used before *L. monocytogenes* infection, and one group of mice (*n* = 9) received intraperitoneal injection of etanercept (30 mg/kg) on days −4, −2, 0, and 3 before and after infection. Controls were treated with KLH (*n* = 9 mice) or PBS (*n* = 10) on the same time schedule as TNFKi vaccination or etanercept treatment, respectively. All groups and TNF^−/−^ mice (*n* = 10) underwent intraperitoneal injection on day 0 with 10^4^ CFU *L. monocytogenes*. On day 4 postinfection, four mice/group were killed, then livers and spleens were harvested and the number of viable bacteria in organ homogenates was determined by plating serial dilutions on trypticase soy broth agar plates (Biovalley) and incubating overnight at 37°C, followed by counting CFU. Survival during infection was evaluated until day 11 (*n* = 5–6 mice/group of treatment).

### ELISA for Anti-KLH and Anti-TNF Antibodies in Serum

Blood samples were collected at different times in accordance with the duration of experiments: 19 and 41 days after the third TNFKi vaccination in the CIA study; 7 days after the third and fourth TNFKi vaccination in the *Listeria* study; 7 days after the third and 32 days after the fourth TNFKi vaccination in the *Mycobacterium* study. Serum was obtained and tested for anti-KLH and anti-TNF-α antibody content. Specific anti-mTNF-α and anti-KLH antibody content was determined by direct ELISA. Precoated ELISA plates with 50 ng per well mTNF-α (PeproTech) or KLH (Sigma) were incubated with serial dilutions of serum from immunized and control mice. Specific antibodies were detected by using phosphatase alkaline-conjugated rabbit anti-mouse IgG (IgG-PA; Sigma-Aldrich A1902). Substrate PNP (Sigma-Aldrich) was added for 40 min. The optical density was measured at 405 nm.

### Tissue Preparation for Histology and Immunohistochemistry

Lungs and livers from *M. tuberculosis*-infected mice collected on days 25–28 and 56 were fixed in 4% buffered formalin and paraffin-embedded, and 4 µm sections were stained with hematoxylin and eosin (H&E) or underwent the Ziehl–Neelsen coloration. Sections were observed by light microscopy at 400× magnification with a Zeiss Observer D1 microscope equipped with a motorized stage connected to Histolab and Archimed software (Microvision, Les Ulis, France). The number and size of granulomas were quantified by using Archimed.

For immunohistochemistry, lung and liver sections were fixed in acetone–ethanol (5 min), and endogenous peroxidase activity was blocked with PBS and 3% H_2_O_2_. Endogenous biotin in the liver was blocked by using an avidin-biotin kit (Vector Laboratories, SP-2001). Tissue sections were incubated for 2 h at room temperature or overnight at 4°C with the primary antibody CD45R (AbD Serotec, MCA1258G) or inducible nitric oxide synthase (iNOS; Abcam, 15323). Sections were then incubated for 30 min at 37°C with the appropriate biotinylated secondary antibody. Avidine biotin peroxidase complexes were added to the sections for 30 min (ABC Vector kit; Vector Laboratories, PK-6100), then incubated with diaminobenzidine substrate (Imm PACT DAB, Vector: SK-4105, Biovalley). After a rinsing in water, sections were counterstained with hematoxylin before mounting and examined by use of a Zeiss Observer D1 microscope as described previously.

For *Listeria* infection, four mice per group were euthanized on day 4 postinfection. Livers were treated as described previously; H&E-stained sections were observed by light microscopy and images were obtained by using a nanozoomer. The number and size of microabcesses were assessed by using NDPview (Hamamatsu, Japan). Immunohistology of livers, macrophages (F4/80, AbD Serotec, MCA497GA), and neutrophils (Ly6G, Abcam, 25377) infiltration was performed as described for CD45R and iNOS staining.

### Statistics

For arthritis studies, serial measurements of clinical variables were analyzed by the area under the receiver operating characteristic curve for each mouse as a summary measure, then as raw data for inter-group comparisons (ANOVA). Differences in arthritis onset were analyzed by ANOVA. Posttest data were compared by data distribution (Student–Newman–Keuls). For *Listeria* infection studies, evaluation of survival involved the Kaplan–Meier method (log-rank test), and bacterial burden data were analyzed by one-way ANOVA with Newman–Keuls posttest. *p* < 0.05 was considered statistically significant.

### Study Approval

All applicable international, national, and/or institutional guidelines for the care and use of animals were followed. For arthritis models, all procedures were approved by the Animal Care and Use Committee of the University of Paris 13 (ethical approval ID: Ce5/2010/036). Infectious protocols were approved by the Ethics Committee for Animal Experimentation of CNRS Campus Orleans (CCO) (no. CLE CCO 2015-1071). We were particularly concerned by a strict application of the 3R rules (18).

## Results

### TNFKi Reduced Clinical and Histological Signs of Arthritis

**Figure 1:**
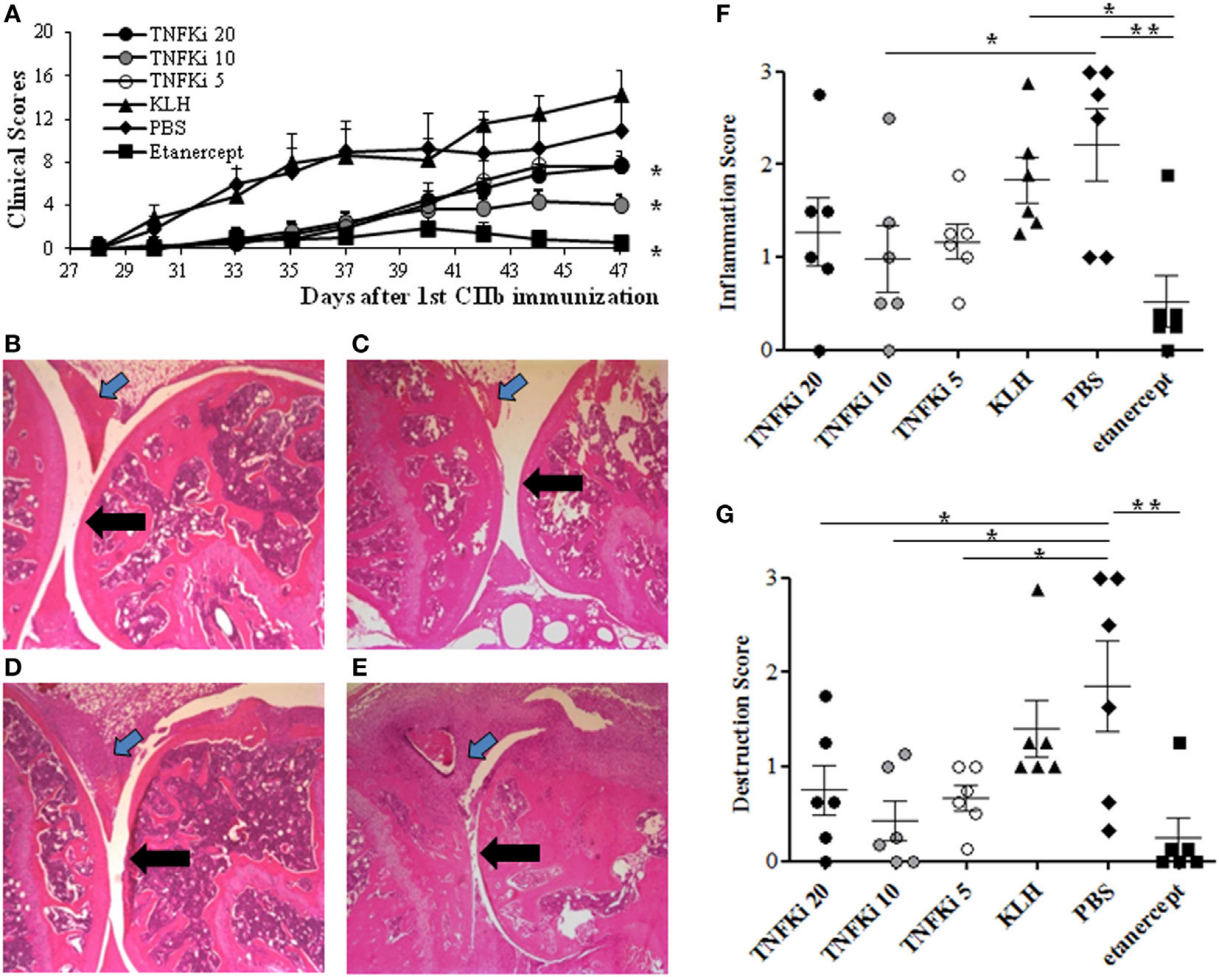
TNFKi protects mice against clinical and histological arthritis. DBA/1 mice were vaccinated three times with keyhole limpet hemocyanin (KLH) or differents doses of TNFKi (20, 10, 5 µg) at days −21, −7, 7 before and during collagen-induced arthritis (CIA) induced by two injections of CIIb (50 μg/mice; day 0 and 21). Treatment with etanercept (30 mg/kg, two times/week) or phosphate-buffered saline (PBS) began from day 22. **(A)** Clinical arthritis was measured by using the sum of arthritis scores (0–4) from four paws (*n* = 6/group). Data are mean ± SEM. *p < 0.05 vs. both PBS and KLH during the whole experiment by ANOVA. The area under the curve (AUC) of clinical scores for each animal are the raw value. **(B–E)** Histology of paws for treatment groups. **(B)** TNFKi-20, **(C)** TNFKi-10, **(D)** TNFKi-5 (20, 10, 5 µg, respectively), **(E)** KLH shows inflammatory synovitis (blue arrow) and articular surface (black arrow). **(F)** Histological inflammation score and **(G)** joint destruction in treatment groups. Horizontal bar is mean, outer bars are SEM, and whiskers are range. *p < 0.05 for TNFKi and **p < 0.01 for etanercept vs PBS (Newman–Keuls test with subsequent *post hoc* comparisons). CIA inhibition with TNFKi was observed in three independent experiments.

To investigate the anti-inflammatory effect of TNFKi *in vivo*, we used the CIA model with DBA/1 mice. Mice were vaccinated three times with 5, 10, or 20 μg/mice TNFKi (TNFKi-5, TNFKi-10, and TNFKi-20 groups). Control groups were vaccinated with KLH or PBS. Etanercept was a positive control. The first signs of arthritis appeared in all control groups at 33 ± 2 days, as expected, but in TNFKi groups at 38 ± 2 days and in the etanercept group at 41 ± 3 days. All anti-TNF treatments resulted in significantly lower clinical arthritis scores as compared with controls (Figure 1A).

Maximal clinical arthritis index (Amax) was significantly lower for mice with 10 µg TNFKi or etanercept treatment than other mice (Table S1 in Supplementary Material). Despite this, the incidence and onset of arthritis were not significantly delayed with TNFKi or etanercept treatment as compared with KLH and PBS controls (Table S1 in Supplementary Material). The weights of TNFKi-vaccinated mice were comparable to that of PBS and KLH controls during the experiment (data not shown).

Histology of paws of PBS and KLH control mice showed significant inflammation, with cell infiltration associated with cartilage destruction (Figures 1B–E). By contrast, TNFKi-10 or etanercept treatment significantly reduced the inflammatory signs of arthritis (Figure 1F). TNFKi-10 or etanercept treatment protected against joint destruction (Figure 1G). In addition, histology showed protection of joints with TNFKi vaccination in the CAIA arthritis model in C57Bl/6 mice (Figure S1 in Supplementary Material).

Thus, TNFKi was effective in protecting against TNF-mediated arthritis in mice.

### TNFKi Did Not Impair Defense to *L. monocytogenes* as Compared with Etanercept

Mice were vaccinated on days −44, −31, −17, and −4 with TNFKi or intraperitoneally injected with etanercept on days −4, −2, 0, and 3 before (or after) infection with *L. monocytogenes*. Controls received PBS or KLH, and TNF^−/−^ mice were a genetic control. All mice were injected intraperitoneally with *L. monocytogenes* (10^4^ CFU) on day 0. Blood was collected before (day −10) and during infection (day 3) to evaluate anti-TNF-α antibody levels. ELISA revealed the production of anti-TNF-α and anti-KLH antibodies in serum from mice with TNFKi vaccination, with an increase in level in responder mice between day −10 and day 3 (Figure S2 in Supplementary Material).

**Figure 2:**
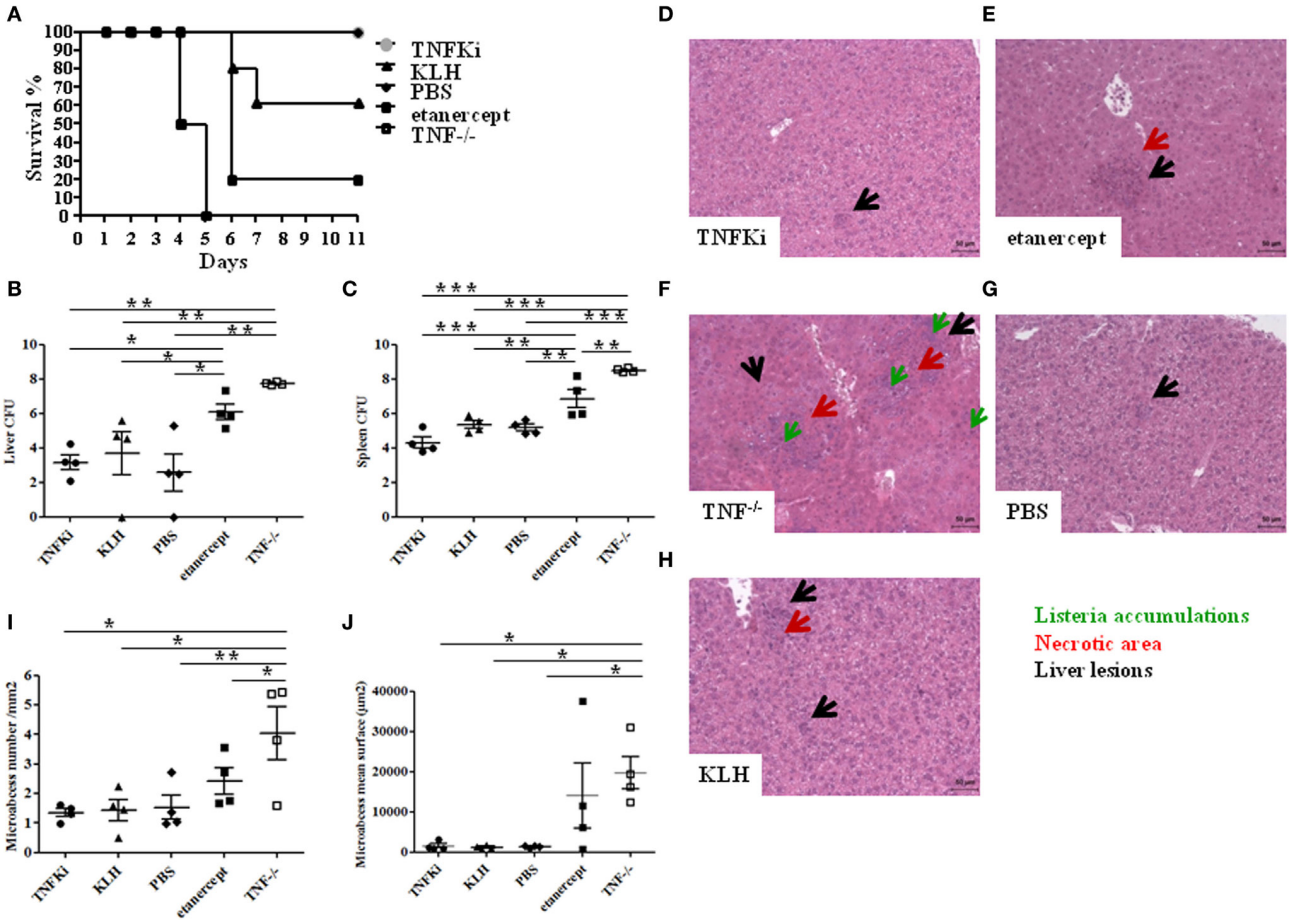
TNFKi vaccination does not alter survival, organ infiltration, and liver lesions during *Listeria monocytogenes* infection. Mice (*n* = 10 per group) were treated with TNFKi or keyhole limpet hemocyanin (KLH) (at days −44, −31, −17, −4) or given phosphate-buffered saline (PBS) or 30 mg/kg etanercept (days −4, −2, 0, and 3). TNF^−/−^ mice were a genetic control. All mice were infected with 10^4^ colony forming units (CFU) *Listeria* at day 0. 4 mice per group were euthanized for bacterial burden and histological analysis at day 4. Survival was evaluated for 11 days postinfection with the remaining mice **(A)**. All TNF^−/−^ mice (*n* = 6) died at 5 days postinfection. Four etanercept-treated mice died on day 6 (*n* = 5) and two mice treated with KLH (*n* = 5). All mice treated with TNFKi (*n* = 6) and PBS (*n* = 6) survived during this period. Survival of TNF^−/−^ and etanercept groups was significantly reduced as compared with PBS, TNFKi, and KLH mice (*Logrank test for trend, p* < 0.0001). Colony-forming units (CFUs) evaluated in liver **(B)** and spleen **(C)** 4 days after infection in mice (*p < 0.05; **p < 0.01, ***p < 0.001, ANOVA). Liver lesions were studied 4 days after infection with *Listeria* after staining sections with hematoxylin-eosin. Liver sections **(D–H)** showing size and number of lesions (black arrow), with necrotic areas (red arrow) and *Listeria* accumulation (green arrow). Quantification of hepatic lesion number **(I)** and surface area **(j)** in slides involved use of NDPview software (Hamamatsu, Japan) (*p < 0.05, **p < 0.01, ANOVA).

Survival of mice was monitored for 11 days postinfection. All TNF^−/−^ mice died within 5 days, and 80% of mice with etanercept treatment died by day 6, as expected; by contrast, mice vaccinated with TNFKi did not die due to infection during this period (Figure 2A). Hence, unlike etanercept, TNF-α neutralization with TNFKi protected against death due to *L. monocytogenes* infection, with a clear survival benefit. The increase in bacterial load in the liver and spleen homogenates seen on day 4 in TNF^−/−^ mice and those with etanercept treatment was not present in either organ of TNFKi-vaccinated mice or in KLH or PBS controls (Figures 2B,C).

We further investigated histological changes in mouse livers on day 4 postinfection (Figures 2D–H). Etanercept-treated and TNF^−/−^ mice showed typical and abundant hepatic microabscesses (Figures 2E,F) characterized by diffuse infiltration and necrotic areas, whereas liver microabscesses in mice vaccinated with TNFKi appeared smaller and similar to those from KLH and PBS control mice (Figures 2D,G,H). Hepatic microabscesses were significantly larger for TNF^−/−^ mice than other groups, except with etanercept treatment (Figure 2J). In addition, the number of microabcesses was significantly larger in TNF^−/−^ mice (Figure 2I), with no difference among TNFKi, KLH, and PBS groups.

**Figure 3:**
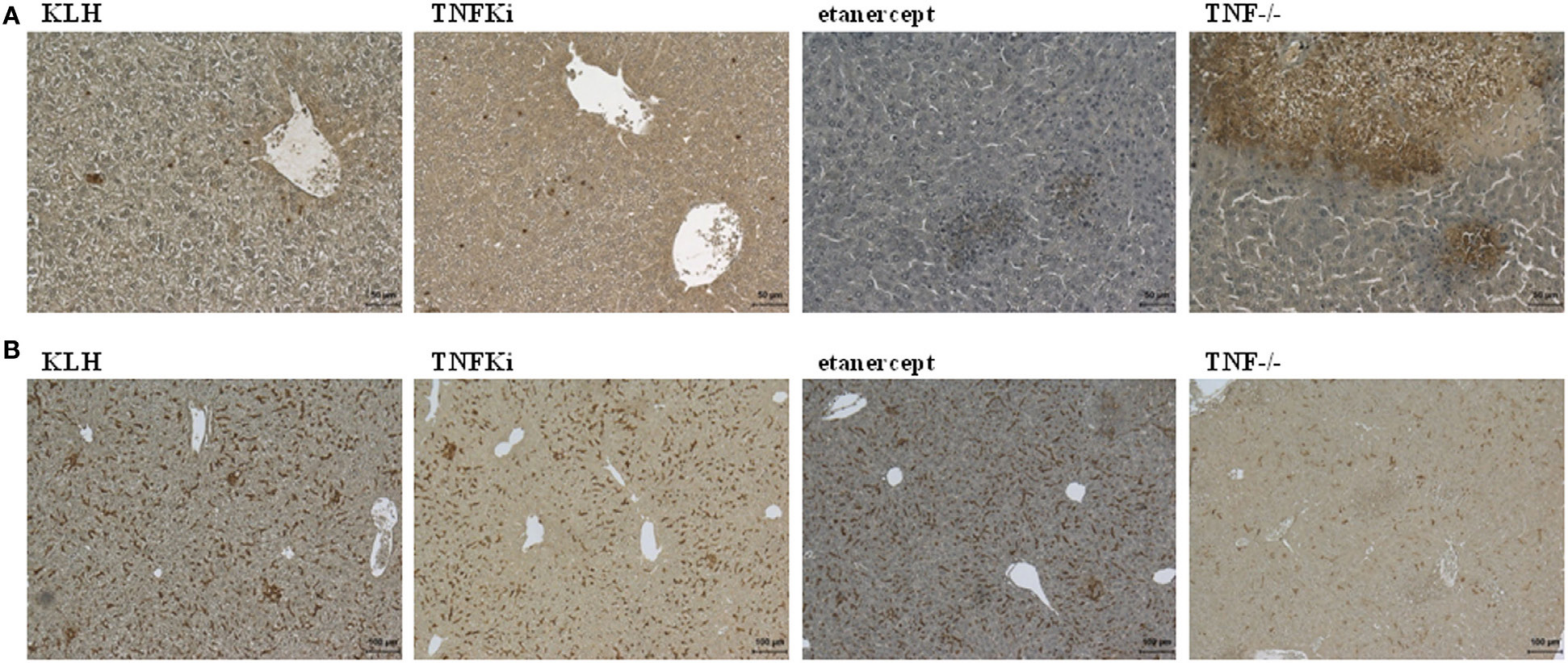
TNFKi vaccination does not alter neutrophils and macrophages infiltration of livers during *Listeria monocytogenes* infection. Immunohistochemical staining of neutrophils expressing Ly6G **(A)** and macrophages expressing F4/80 **(B)** after 4 days of infection [scale bar **(A)** 50 µm; scale bar **(B)** 100 µm].

Immunohistochemistry of liver sections to investigate the cellular composition of microabcesses after 4 days of infection revealed an increased accumulation of neutrophils (Ly6G) in TNF^−/−^ and etanercept-treated mice but not TNFKi, KLH, and PBS mice (Figure 3A). Macrophage staining (F4/80) appeared more dispersed and less localized inside microabcesses in etanercept-treated and TNF^−/−^ mice as compared with TNFKi, KLH, and PBS treatment (Figure 3B).

### TNFKi Did Not Affect the Defense against *M. tuberculosis* Infection

We assessed the consequences of TNFKi vaccination during early and established *M. infection*. Mice were vaccinated on days −44, −31, −17, and −4 with TNFKi (10 μg/mice) or treated with etanercept (30 mg/kg twice a week from day −4 to day 52 before or after infection). Control groups were mice with KLH vaccination or PBS treatment. TNF^−/−^ mice, which are very sensitive to virulent *M. tuberculosis* H37Rv infection, were used as a positive control of total infection in the absence of functional TNF. Except for five naïve mice, all mice were infected on day 0 by intranasal instillation of *M. tuberculosis* H37Rv (2,250 CFU). TNF^−/−^ mice lost weight rapidly and had to be euthanized on day 25 postinfection, but the other mice did not experience body weight loss (data not shown) and were euthanized during early infection (day 28) or established infection (day 56).

Blood was collected before (day −10) and during infection (day 28) to evaluate levels of anti-TNF-α antibodies. As expected, ELISA revealed a sustained serum production of anti-TNF-α antibodies in mice vaccinated with TNFKi (Figures S3A,B in Supplementary Material).

**Figure 4:**
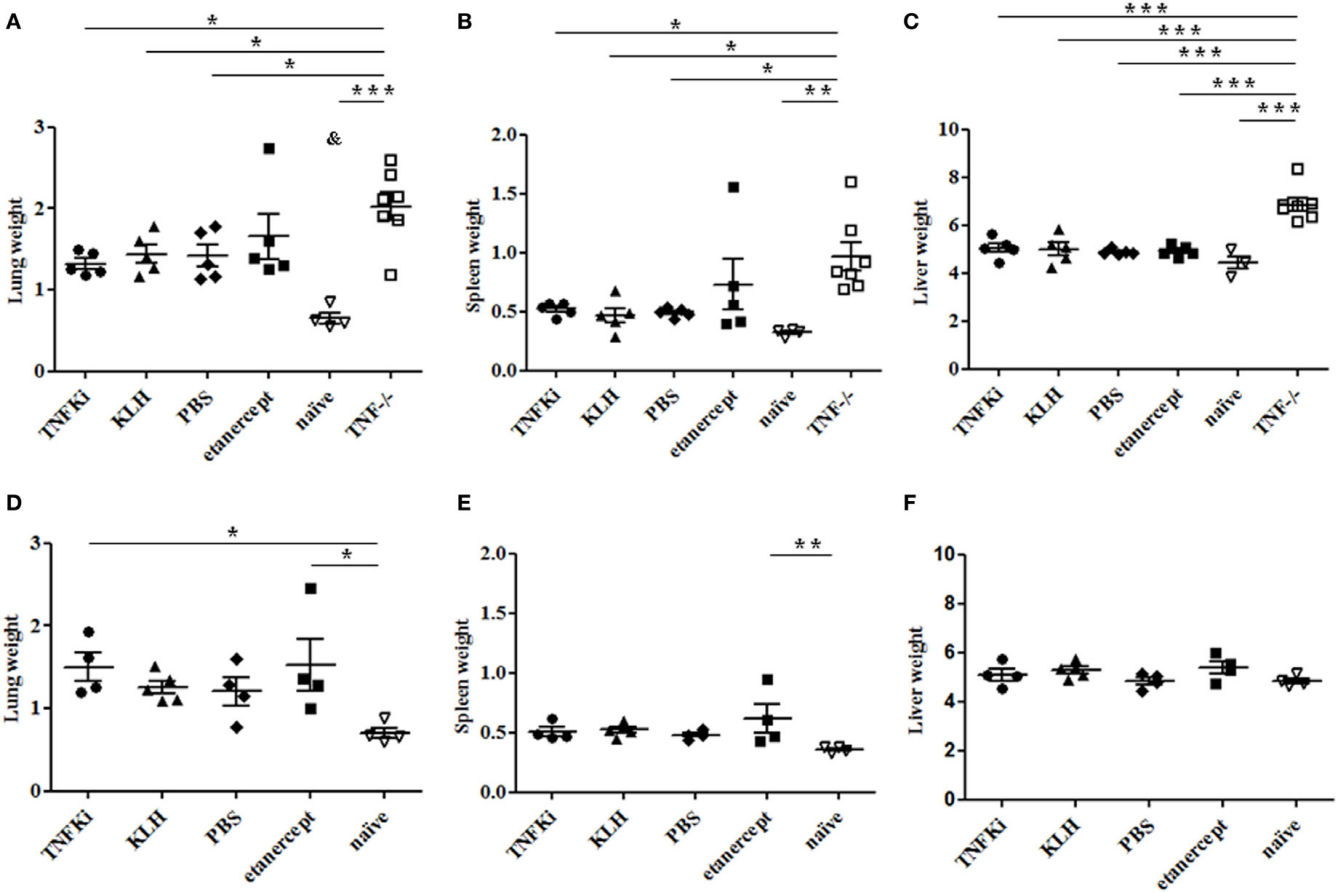
Organ weights during early and established *M. tuberculosis* infection. C57Bl/6 mice were vaccinated with keyhole limpet hemocyanin (KLH) (*n* = 10) or 10 µg TNFKi (*n* = 10) at days −44, −31, −17, and −4 before infection. One group of mice received 30 mg/kg of etanercept (*n* = 10) twice a week from day 4 to day 52. Except for naïve mice (*n* = 5), all treated mice and TNF^−/−^ mice (*n* = 7) were infected intranasally with 2,250 CFU of *M. tuberculosis* on day 0. Five mice per treatment (day 28) and all TNF^−/−^ mice (day 25) were euthanized and lung, spleen, and liver were weighed. Survival was monitored until day 56 (4–5 mice/group) and organs were weighed at euthanasia. Relative organ weights were calculated as the ratio of absolute organ weights to body weights. Organ weights of mice during early infection **(A–C)** and established infection **(D–F)**. Organ weights were expressed as % of body weight. Horizontal bar is mean, outer bars are SEM, and whiskers are range. *p < 0.05 vs TNFKi, KLH, phosphate-buffered saline (PBS), ***p < 0.001 vs naïve mice; Newman–Keuls test with *post hoc* comparisons. ^&^*p* < 0.05 naïve vs TNFKi, KLH, PBS, and etanercept.

At euthanasia, weights of lungs, spleens, and livers were evaluated as indicators of inflammation. During early infection, all TNF^−/−^ mouse organs showed a marked increase in weight as compared with the other groups, with the exception of lung and spleen of etanercept-treated mice (Figures 4A–C). At day 56, established infection, organ weights were similar between all infected mice (Figures 4D–F).

**Figure 5:**
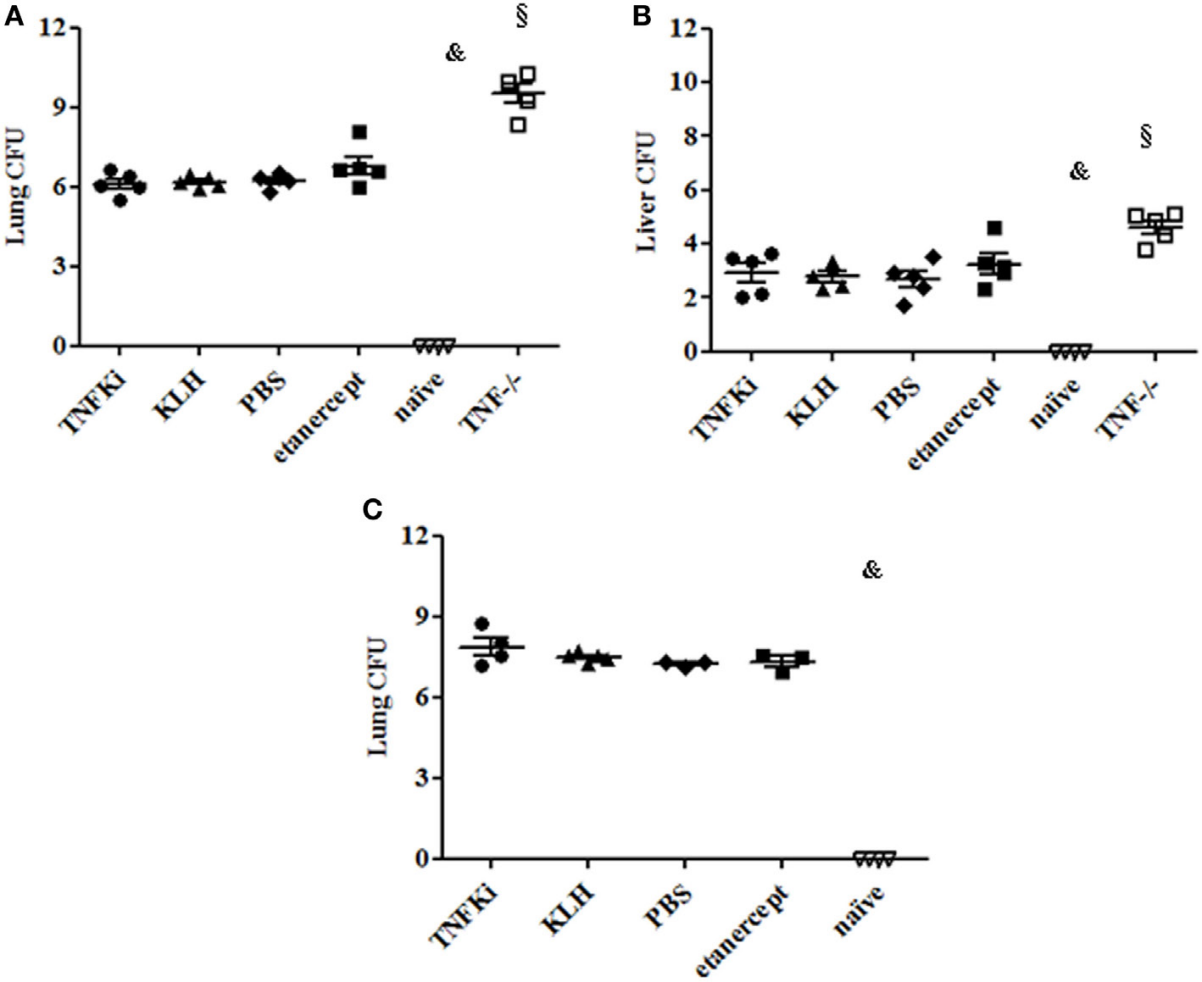
TNFKi did not increase bacterial loads in lung and liver of *Mycobacterium tuberculosis*-infected mice. Vaccination schedule described previously. During early **(A,B)** and established infection **(C)**, a part of lungs and liver were weighted and used after lysis for bacterial culture and colony formation units (CFU) evaluation. Results are expressed as Log_10_ of CFU count per organ. CFU number in organs of mice during early infection **(A,B)** and lung during established **(C)** infection. Horizontal bar is mean, outer bars are SEM, and whiskers are range (^§^*p* < 0.001 TNF^−/−^ vs all groups; Newman–Keuls test with subsequent *post hoc* comparisons).

Liver and lung bacterial loads were then assessed in vaccinated and TNF^−/−^ and etanercept-treated mice. During early infection, total CFU were greatly increased in organs of TNF^−/−^ mice as compared with all other groups (^§^*p* < 0.001 TNF^−/−^ vs all groups in lung and liver, ANOVA) (Figures 5A,B). At the same time, CFU per organ were similar between TNFKi, etanercept, and control groups (KLH and PBS). At day 56, established infection, total CFU were similar in lungs of all infected mice (Figure 5C).

**Figure 6:**
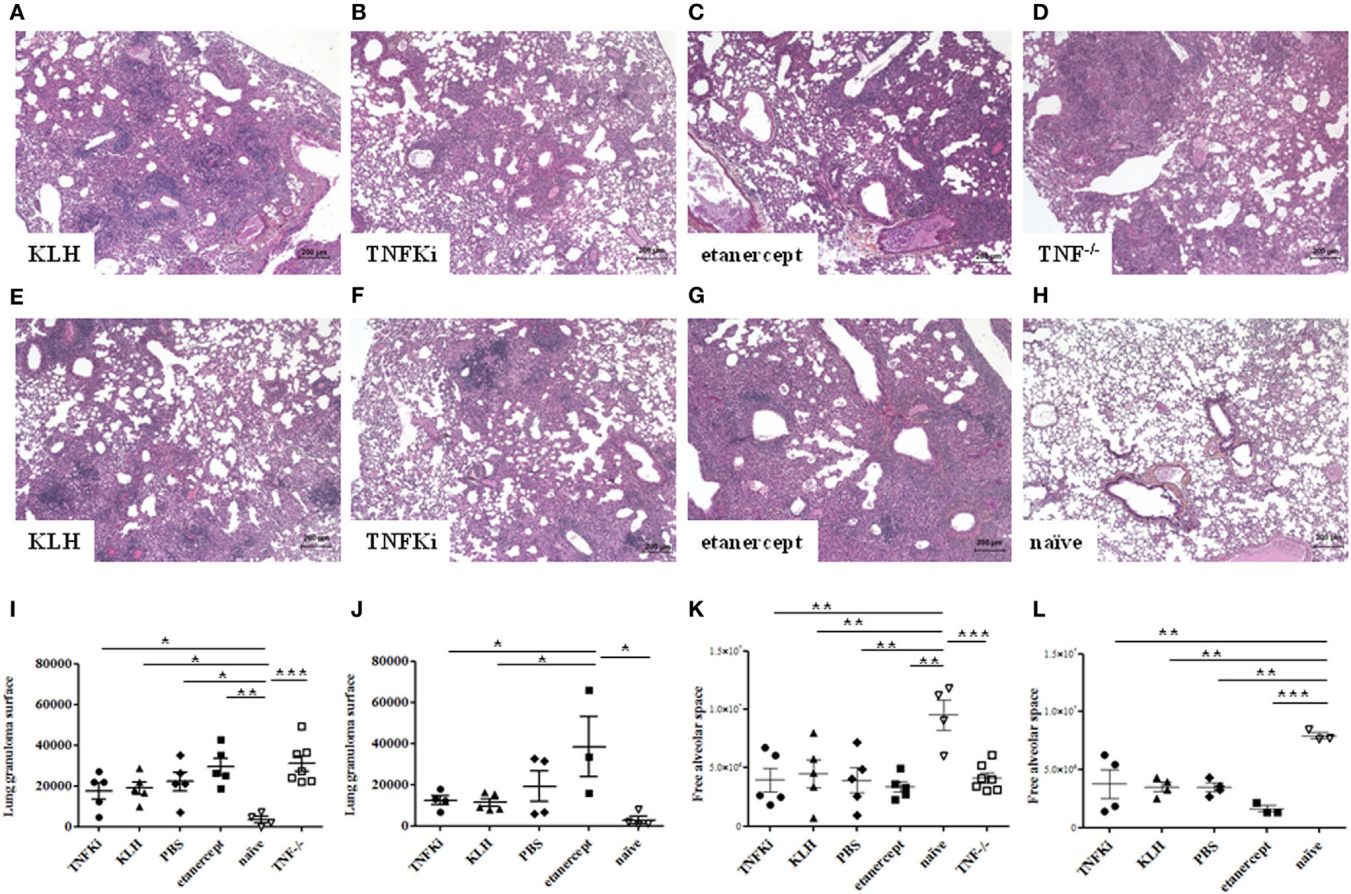
Limited effects of TNFKi on lung granuloma formation during *Mycobacterium tuberculosis* infection. Granulomas were assessed during early **(A–D)** and established (**E–H**) infection. Lung sections were stained with hematoxylin-eosin (*n* = 3–7 per group). Granulomas in lungs during early infection **(A–D)** and established infection **(E–H)**. Quantification of lung granuloma surface area during early **(I)** and established infection **(J)** and alveolar space of lungs during early **(K)** and established infection **(L)**. Horizontal bar is mean, outer bars are SEM, and whiskers are range (*p < 0.05, **p < 0.01, ***p < 0.001, ANOVA).

To determine the effect of TNFKi on organ integrity during early and established infection, we investigated histological changes in mouse lung and liver. Indeed, during early infection, TNF^−/−^ mice showed inability to control *M. tuberculosis* infection, with rapid, exacerbated lung inflammation, and many granulomas with large necrotic areas (Figure 6D). In the lungs of TNFKi, KLH, and PBS groups, granulomas were more discrete, with the typical granuloma structure at day 56 (Figures 6A–H). Etanercept-treated mice showed granulomas on day 28, but these were more developed and less structured by day 56 (Figures 6C,G). With Archimed software quantification, during early infection, the lung granuloma surface area was increased but not significantly in TNF^−/−^ and etanercept-treated mice (Figure 6I). At day 56, the granuloma surface area of TNFKi- and KLH-vaccinated mice appeared decreased, contrary to etanercept treatment (*p < 0.05 vs etanercept group, ANOVA) (Figure 6J). Granuloma numbers were similar between mouse groups at both phases (data not shown). During both early and established infection, all infected mice showed less free alveolar space as compared with naïve mice (Figures 6K,L), which was most prominent with etanercept treatment on day 56 (Figure 6L).

**Figure 7:**
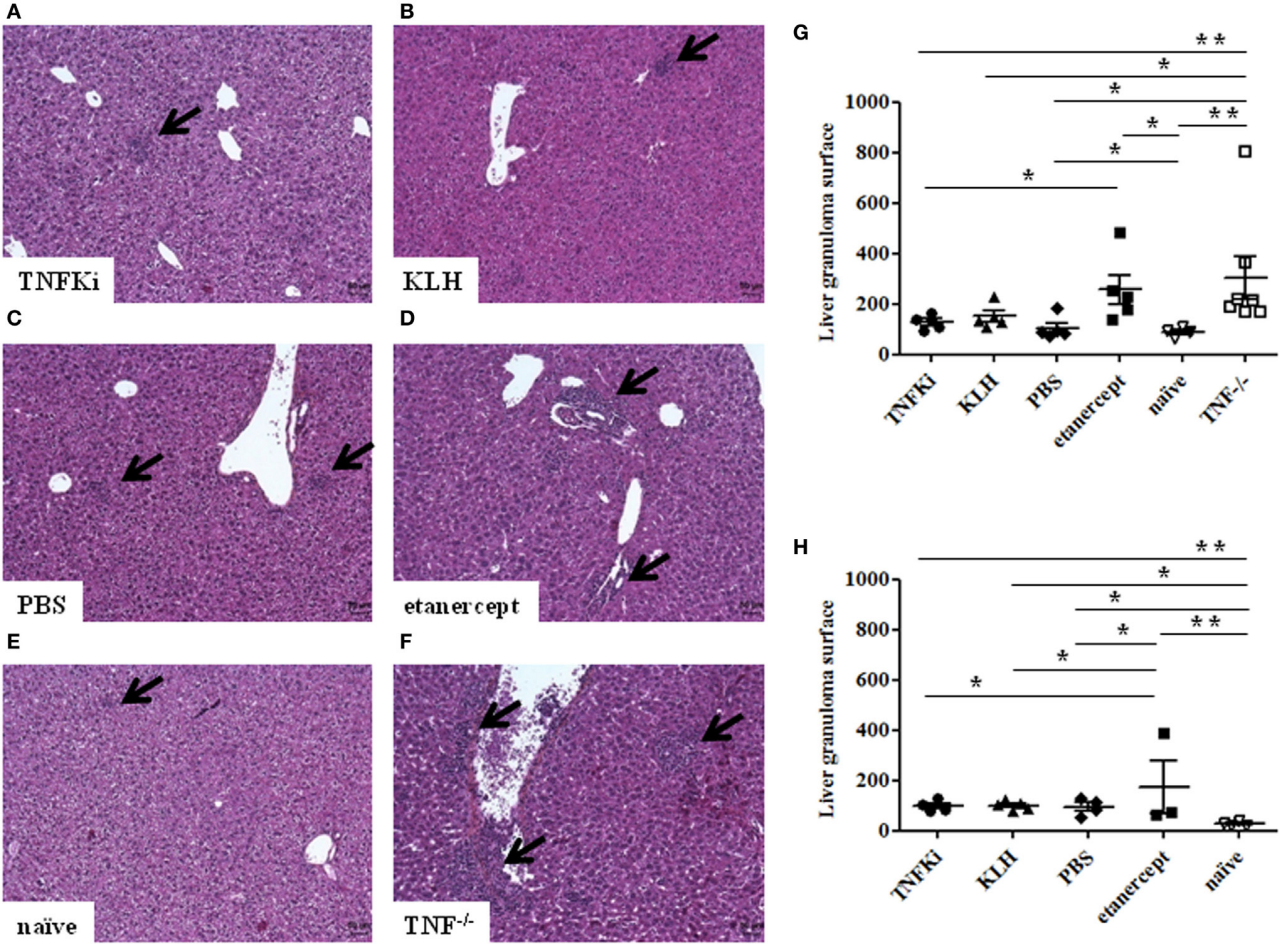
TNFKi did not induce larger granuloma formation in liver of *Mycobacterium tuberculosis-*infected mice. Histology of granulomas (arrows) in liver sections stained with hematoxylin-eosin (*n* = 3–7 per group) during early infection **(A–F)**. Quantification of hepatic granuloma surface area during early **(G)** and established infection **(H)**. Horizontal bar is mean, outer bars are SEM, and whiskers are range (*p < 0.05, **p < 0.01, ANOVA).

Histology of the liver at the early phase of infection (Figures 7A–F) revealed that granuloma surface area was significantly larger for TNF^−/−^ and etanercept-treated mice than other mice (Figure 7G) (*p < 0.05 vs TNFKi).

TNF^−/−^ and etanercept-treated groups showed a higher number of granulomas, although not significantly (data not shown). At day 56, liver granuloma surface area was larger for etanercept-treated mice than other groups (Figure 7H). Hence, histology of both lungs and livers suggested that mice vaccinated with TNFKi, similar to KLH or PBS, effectively controlled the infection during the progression of the disease as compared with etanercept.

**Figure 8:**
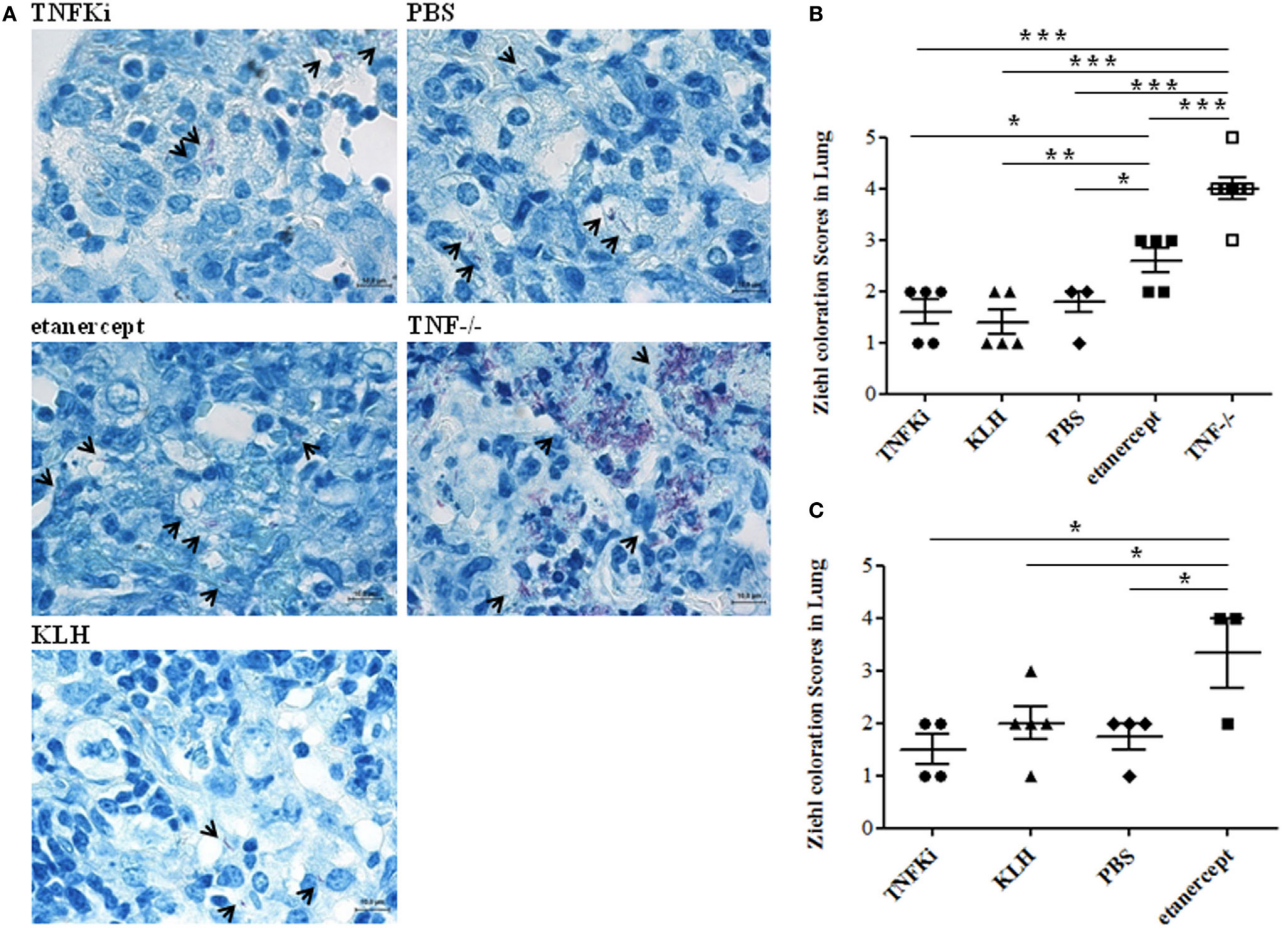
TNFKi did not increase bacterial load in lungs of *Mycobacterium tuberculosis*-infected mice. Ziehl–Neelsen coloration showing direct recognition of mycobacteria in lung sections. *M. tuberculosis* appeared as red bacilli (arrowheads). Examples of images at early phase of infection **(A)**. Quantification of mycobacteria during the early **(B)** and established infection **(C)** after blinded observations of three fields per mice (*n* = 3–7 mice per group) (*p < 0.05 ANOVA). Scale bar, 10 µm.

To confirm mycobacterial infiltration in granulomas, we used Ziehl–Neelsen coloration and semiquantitative evaluation of bacterial staining in mouse lungs (Figure 8). Microscopy revealed TNF^−/−^ and etanercept-treated mice with greater mycobacteria infiltration than other groups during early and established infection (Figures 8B,C, *p < 0.05 for etanercept and ***p < 0.001 for TNF^−/−^ mice vs TNFKi, KLH, PBS, ANOVA).

We used immunohistochemistry of mouse lung sections to investigate the relationship between cellular infiltration or activation status and TNF-α neutralization. During early infection, iNOS was expressed in granulomas of all mouse groups (Figure S4A in Supplementary Material). Histology revealed lesions in TNFKi, KLH, and PBS lungs characterized by macrophage infiltration and a very low number of neutrophils. Conversely, in TNF^−/−^ and etanercept-treated lungs, iNOS expression in macrophages co-localized with microabscesses containing altered neutrophil number. Etanercept-treated lungs showed more macrophage infiltration, higher number of foamy histiocytes expressing iNOS, and fewer neutrophils during established than early infection (Figures S4A,B and S5 in Supplementary Material). Lesions of PBS-treated lungs showed few neutrophils, and iNOS was expressed in macrophages and foamy histiocytes. Lesions of mouse lungs with KLH or TNFKi vaccination showed similar cellular infiltration as for PBS-treated lungs but a lower number of foamy histiocytes. Thus, macrophages within the granuloma of TNFKi-vaccinated lungs retained the bactericidal function controlling infection.

**Figure 9:**
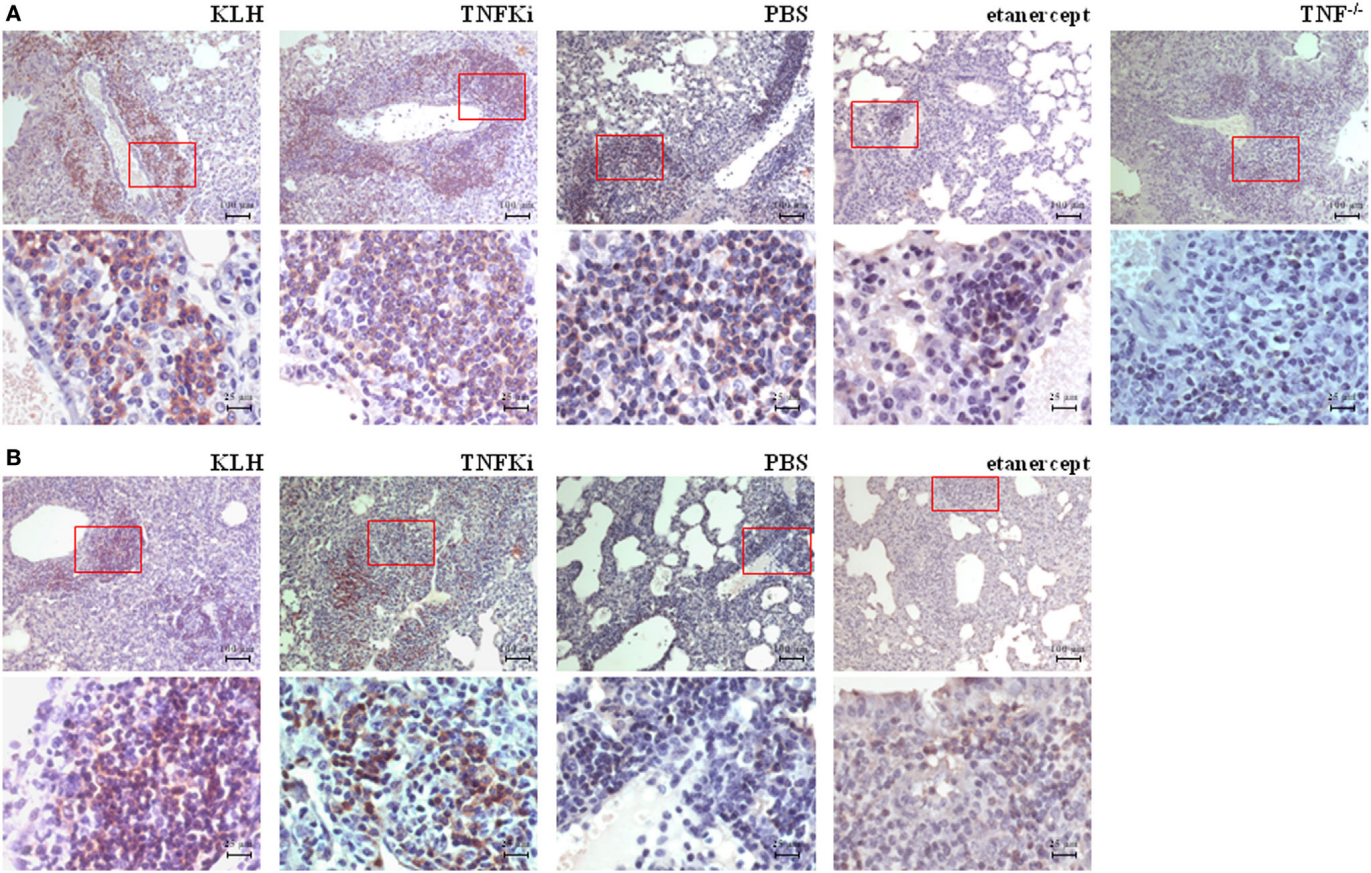
TNFKi favored B-lymphocyte infiltration in lungs of *Mycobacterium tuberculosis*-infected mice. Immunohistochemical staining of B lymphocytes expressing CD45R. During early infection **(A)** and established infection **(B)**, perivascular accumulation of B lymphocytes in lungs of mice. Representative images of 3–7 mice per group. Scale bar, 100 µm. 4× magnification of red boxes below original picture (scale bar, 25 µm).

We explored the effect of the TNFKi on lung infiltration by B lymphocytes during *M. tuberculosis* infection (Figure 9). During early infection, anti-CD45R staining revealed a massive infiltration of B lymphocytes in lungs of TNFKi- and KLH-vaccinated mice, lower infiltration with PBS, and a near absence of B lymphocytes in TNF^−/−^ or etanercept-treated mice. Immunostaining revealed a higher perivascular accumulation of B cells with TNFKi or KLH vaccination. During late infection, with TNFKi or KLH vaccination, pulmonary granulomas were well organized, characterized by many pulmonary nodules and accumulation of B lymphocytes. The phenotype for PBS lungs was similar to that for vaccinated lungs but with fewer B lymphocytes. By contrast, etanercept treatment resulted in massive macrophage infiltration with a large necrotic area, without typical granulomas and few B lymphocytes (Figure 9).

Thus, TNFKi vaccination did not impair the host control of *M. tuberculosis* infection in terms of lung and liver bacterial burden, granuloma formation or inflammatory response as compared with TNF neutralization with etanercept.

## Discussion

Here, by using a surrogate of human TNF-α vaccine, mouse TNFKi, we validated the anti-inflammatory effect of the vaccine in an experimental model of RA while documenting the little perturbed control of infection in TNFKi-treated mice in two models of infection, with *L monocytogenes* and *M. tuberculosis*.

A major concern of anti-TNF-α treatments is an increased risk of serious bacterial, viral, and fungal infections. Tuberculosis is the most common opportunistic infection and, to a lesser extent, bacterial infections, such as listeriosis (19–21). Anti-TNF-α vaccination has been widely explored in mice (22–24). Clinical trials in humans were promising in the early phases of development of TNF-α vaccination with a TNF-Kinoid (11), although not considered effective at 6 months in a larger study with a single low dose of vaccine that did not result in significant production of anti-TNF-α auto-antibodies (Neovacs.fr/products/tnf-kinoid/, NCT01911234). The TNFKi vaccine used in the present study consisted of mouse TNF-α linked to the KLH carrier protein. TNFKi induced the production of anti-TNF-α polyclonal antibodies and alleviated arthritis in two experimental models of arthritis, CIA and CAIA, which confirms the effective neutralization of TNF-α.

To assess to what extent this novel anti-TNF strategy would affect immunity against infectious agents, we used two experimental models of infection, with *L monocytogenes* and *M. tuberculosis*. In both cases, we compared the effect of TNFKi to that of complete lack of TNF-α (TNF^−/−^ mice) and a current anti-TNF-α treatment (etanercept). Etanercept is considered the safest anti-TNF inhibitor in terms of the possible risk of serious infection (notably tuberculosis reactivation). It is a pharmacological reference for anti-TNF-α blockade in animal models of infection (25). With *L. monocytogenes* infection, anti-TNF-α polyclonal antibody production induced by TNFKi did not impair immunity to the bacterium, and all mice survived. However, TNF-α neutralization with etanercept enhanced the susceptibility to infection and TNF^−/−^ mice did not show resistance to infection. TNFKi-vaccinated mice showed the same bacterial clearance in liver and spleen as immunocompetent mice (PBS and KLH controls), whereas etanercept-treated mice showed increased bacterial load. In agreement, the size and number of liver microabcesses were similar between TNFKi and control mice at day 4 postinfection. In contrast, TNF^−/−^ mice showed increased number of lesions and large lesions characterized by necrotic areas. Immunization with TNFKi did not alter immune cell migration into the liver during *Listeria* infection, whereas etanercept-treated and TNF^−/−^ mice exhibited neutrophil accumulation and reduced macrophage recruitment in lesions.

With the model of *M. tuberculosis* infection, TNF neutralization by TNFKi vaccination did not increase the sensitivity to the pathogen, but TNF^−/−^ mice died during the early phase of infection. Previously, mice treated with a murine homolog of etanercept died due to *M. tuberculosis* infection (26). We did not observe such a drastic effect on survival with etanercept treatment; however, Zielh–Neelssen staining revealed higher bacteria loads in lungs from TNF^−/−^ and etanercept-treated mice than TNFKi-vaccinated mice.

We reported previously and confirm here the striking lung inflammation in TNF^−/−^ mice, including large necrotic granulomas (27), which were not observed in the other groups in our study. Granuloma formation and organization in lungs was not altered with established *M. tuberculosis* infection and TNFKi vaccination as compared with etanercept treatment. In addition, granulomas observed in TNFKi-vaccinated livers were similar to those in control livers. In both TNF^−/−^ and etanercept-treated mice, liver granulomas were significantly larger.

The protective role of iNOS in infection led us to study its expression during the anti-*M. tuberculosis* response. Indeed, mice deficient in iNOS die due to tuberculosis infection within 33–45 days, and iNOS inhibition aggravates the course of murine tuberculosis (28, 29). We found that iNOS was expressed exclusively by macrophages within granulomas during the early phase of infection and by macrophages and foamy histiocytes during established infection. Strikingly, recruitment of macrophages and neutrophils seemed altered by etanercept treatment, because numerous neutrophils and few macrophages infiltrated lungs in the acute phase as compared with numerous macrophages and foamy histiocytes infiltrating lungs in the established phase. With TNFKi treatment, neutrophilic infiltration was rare, and few foamy histiocytes were observed during established infection. The adaptive immune response to *M. tuberculosis* is also mediated by B lymphocytes. Despite low risk of tuberculosis reactivation for RA patients receiving rituximab to deplete B cells (30), a recent study showed that in *M. tuberculosis*-infected macaques, rituximab altered local granulomatous response during acute infection (31). Furthermore, another study showed that TNF-α secreted by B lymphocytes is important for aggregation (32). In our study, contrary to etanercept, TNFKi vaccination did not impair B-cell recruitment. At day 56 postinfection, B cells formed nodules in lungs of all mouse groups, except with etanercept treatment. TNFKi allowed for B-cell recruitment in infected tissues and effective granuloma formation and maintenance during both early and established infection.

Why TNF-α neutralization with anti-TNF vaccination preserved the immune response against infection remains speculative. The risk of infection depends on the nature of the TNF-α blockers. Several studies have shown increased risk of reactivation of latent tuberculosis with infliximab and adalimumab monoclonal antibody therapy than with the recombinant soluble TNF-α receptor etanercept (33, 34). The safety profile with both types of treatments may differ in part due to their TNF-α binding property. Infliximab forms stable complexes with soluble TNF-α and TNF-α expressed at cell membranes, whereas etanercept interacts with soluble TNF-α and only weakly and reversibly with the transmembrane form (35).

Although the importance of soluble versus membrane TNF-α has not been investigated in detail in the CIA model, the role of both forms of TNF-α were well investigated in infectious models. Indeed, mice lacking the soluble form of TNF-α but expressing a transmembrane form or expressing uncleavable membrane TNF showed control of *M. tuberculosis* infection, at least during the acute phase (36–38) or during *L. monocytogenes* infection (39). These latter mice could still form compact granulomas, which prevented bacterial spreading, whereas mice completely lacking TNF-α were severely impaired in granuloma formation. Expression of a functional transmembrane form of TNF-α also confers partial protection against *L. monocytogenes* infection (40), but TNF-α- or TNF-R1-deficient mice are highly susceptible (41).

Low levels of TNF present at the site of infection might be sufficient to control mycobacteria infection. Indeed, in TNF-deficient mice, which usually rapidly die after *Mycobacterium bovis* bacillus Calmette–Guérin (BCG) infection, the antimycobacterial immune response was restored by infection with a TNF-α-secreting recombinant *M. bovis* BCG, effectively protecting these highly susceptible mice (42).

There are some limitations to this work that warrant further studies. The exact mechanisms by which the two different anti-TNF strategies affect the immune response to infectious agents need to be further dissected. Another issue is the extrapolation of the results obtained in the animal model to humans, which is not the aim of the present study. Although we used validated models of arthritis (CIA) and infection, these models only partially mimic human disease. Moreover, differences between the human and murine immune system both in response to vaccination and infection may lead to deep discrepancies that warrant caution. No safety issues or increased risk of infection were reported when anti-TNF vaccination was administered in humans (Crohn’s disease: NCT00808262; RA: NCT01040715). Nevertheless, the samples of those trials were limited and no specific studies on response to infection were performed.

In summary, arthritis improvement when targeting TNF-α does not result in a constant and uniform alteration of the host defense against infection. This alteration is variable depending on the strategies used for targeting TNF-α. TNFKi may allow for maintenance of the host defense against *M. tuberculosis* and *L. monocytogenes* infection in mice. This is a major point for the safety features of therapeutic TNF vaccination in chronic inflammatory diseases. Pathogenic levels of TNF-α might be neutralized efficiently while maintaining the minimal levels needed to control host resistance to bacterial infection.

## Ethics Statement

All applicable international, national, and/or institutional guidelines for the care and use of animals were followed. For arthritis models, all procedures were approved by the Animal Care and Use Committee of the University of Paris 13 (ethical approval ID: Ce5/2010/036). Infectious protocols were approved by the Ethics Committee for Animal Experimentation of CNRS Campus Orleans (CCO) (no. CLE CCO 2015-1071). We were particularly concerned by a strict application of the 3R rules.

## Author Contributions

NB performed mice immunization, evaluated clinical arthritis, performed immunoassay, *Listeria* infection, and immuno-histological analysis and helped draft the manuscript. LS performed statistical analysis, drafted and revised the manuscript. NS performed *Mycobacteria* infection, bacterial burden, and organ weight evaluations. DD participated in the immuno-histological analysis, analysis of the data, and helped to draft the manuscript. PD helped coordinate the study and helped draft the manuscript. BR and VQ established and validated the experimental infectious models and conceived a part of the *Listeria* and *Mycobacteria* infection study, participated in coordination and analysis of the data, and helped draft the manuscript. M-CB conceived of the study, analyzed the data, and helped to draft the manuscript. EA conceived of the study, performed coupling of TNF vaccine, participated in coordination of the study, analyzed the data, and drafted the manuscript.

## Conflict of Interest Statement

BR, DD, EA, LS, M-CB, NB, NS, PD, and VQ declare no conflict of interest.
