# Supplementary material for: Tumor Necrosis Factor-Alpha Targeting Can Protect against Arthritis with Low Sensitization to Infection
Source: Front Immunol. 2017 Nov 14;8:1533. doi: 10.3389/fimmu.2017.01533 (PMC5694445; doi:10.3389/fimmu.2017.01533)
Supplement: Supplementary file 1 [file Presentation_1.PDF]

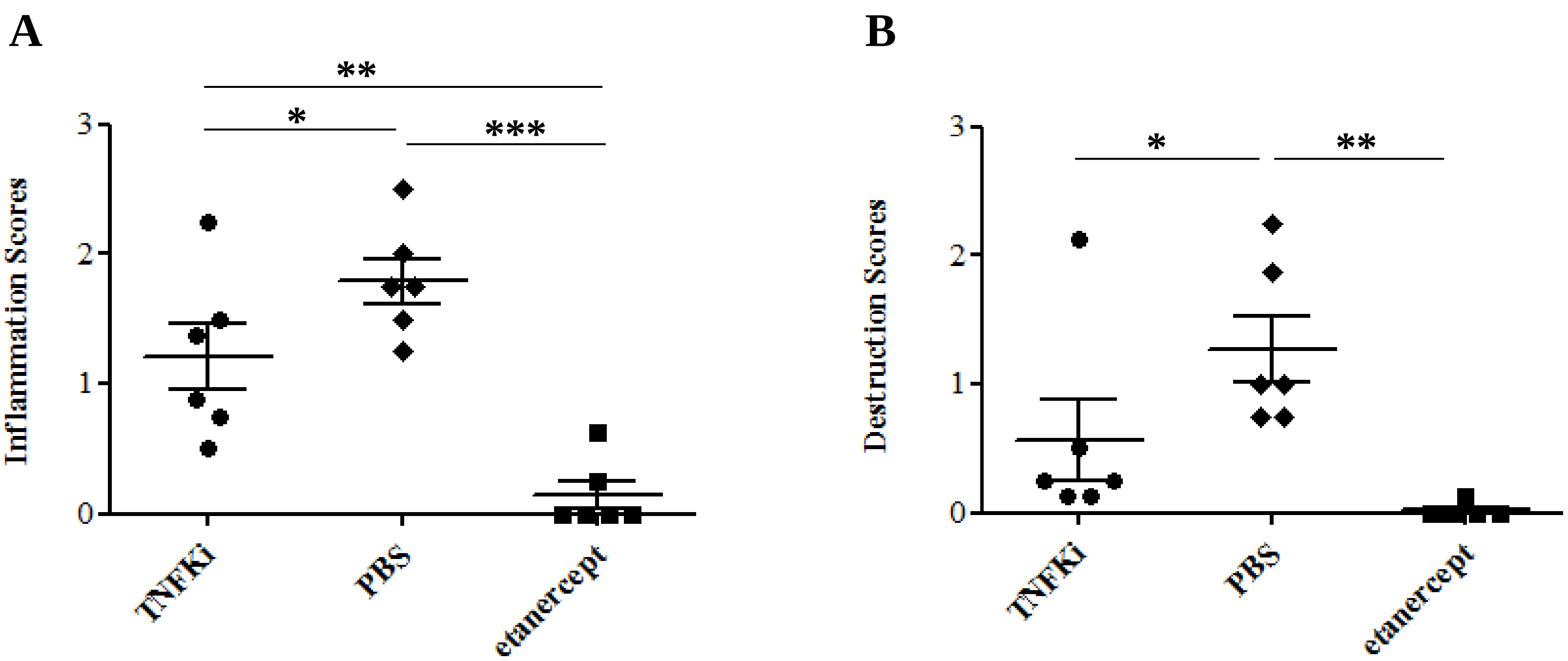

Supplementary figure 1: TNFKi vaccination reduces histological signs of arthritis in the Collagen Antibody-Induced Arthritis model. C57Bl/6 female mice were immunized with 10µg of TNFKi vaccine (n=6) at days -37, -13 and -5. Last groups of mice received 30mg/kg of etanercept (n=6) or PBS (n=6) twice a week from day-5 to day 12. Arthritis was induced by an intraperitoneal injection of a cocktail of five anti-collagen antibodies (5 mg per mice) at day 0 and a boost of 50µg LPS at day 3. Arthritis onset occurs around 3-4 days after anti-collagen antibodies injection and peak around day 7-10. After euthanasia at day 12, histological inflammation (**A**) and destruction (**B**) after staining of knee sections with hematoxylin-eosin. TNFKi vaccinated mice presented lower inflammation and destruction scores than PBS control group (*p<0.05, ANOVA).

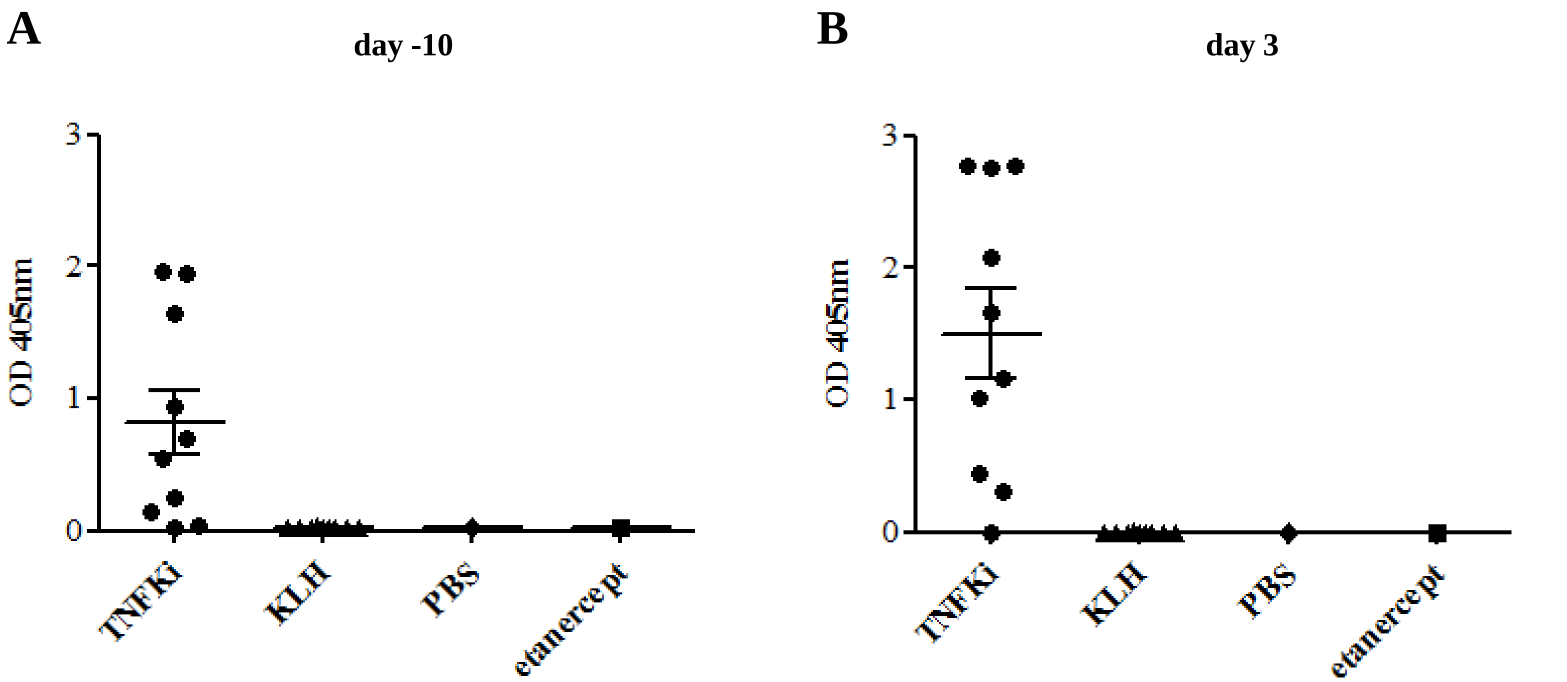

Supplementary figure 2: TNFKi vaccination induces a sustained anti-TNF antibody production during *L. monocytogenes* infection. C57Bl/6 mice were immunized with KLH (n=9) or with 10µg of TNFKi vaccine (n=10) at days -44, -31, -17 and -4. One group of mice received 30mg/kg of etanercept (n=9) or PBS (n=10) at day -4, -2, 0 and 3. All mice and $TNF^{-/-}$ mice (n=10) were infected by intraperitoneal injection with 10^4 CFU of *Listeria monocytogenes* at day 0. Blood was collected at day -10 (**A**) and at early phase of infection at day 3 (**B**) to evaluate the anti-TNF antibodies production. ELISA analysis showed that TNFKi vaccinated mice produced high levels of anti-TNF Abs, with an increase in responder mice between day -10 and day 3.

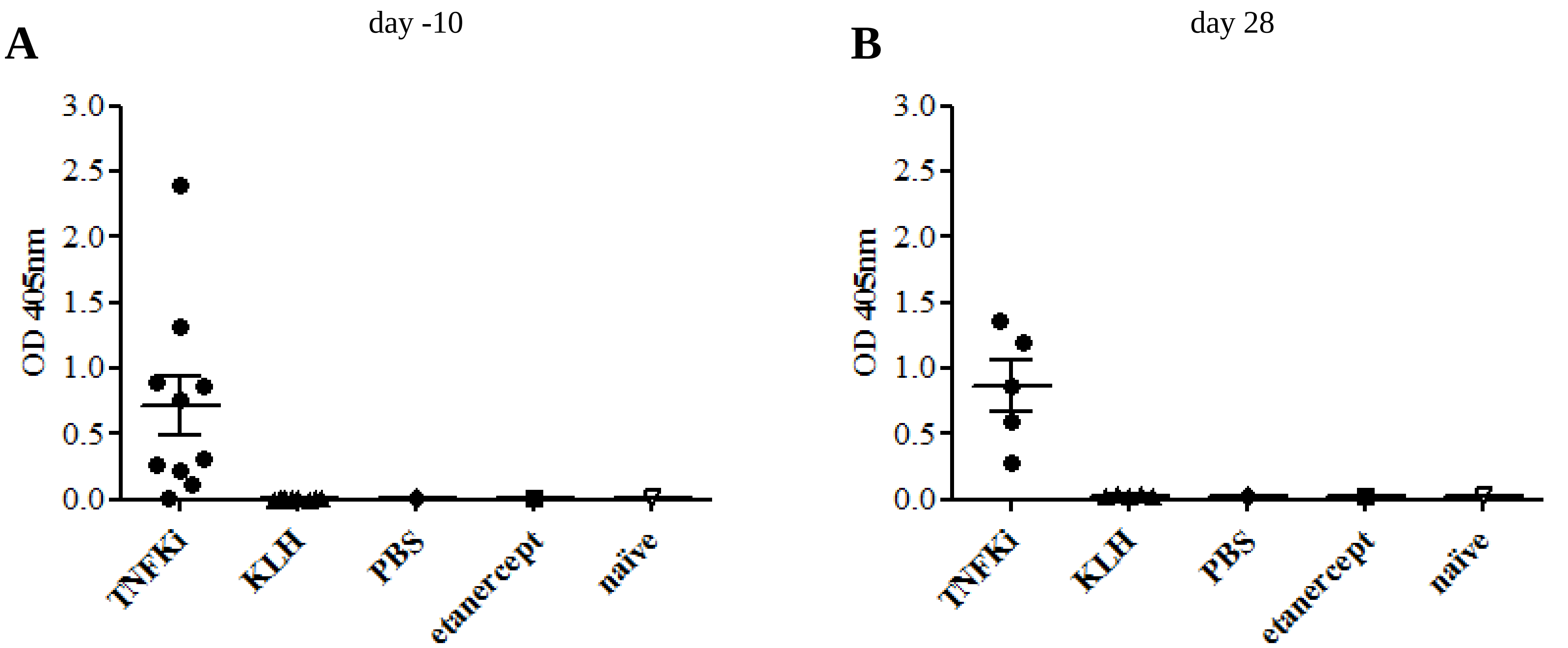

Supplementary figure 3: TNFKi vaccination induces a sustained anti-TNF antibody production during *M. tuberculosis* infection. C57Bl/6 mice were immunized with KLH (n=10) or with 10µg of TNFKi vaccine (n=10) at days -44, -31, -17 and -4. One group of mice received 30mg/kg of etanercept (n=10) twice a week from day-4 to day 52. Excepted naïve (n=5), all mice and $TNF^{-/-}$ mice (n=7) were infected intranasally with 2250 CFU of *M. tuberculosis* at day 0. Blood was collected at day -10 (**A**) and during infection at day 28 (**B**) to evaluate the anti-TNF antibodies production. ELISA analysis showed that TNFKi vaccinated mice produced high levels of anti-TNF Abs at day -10 and 28.

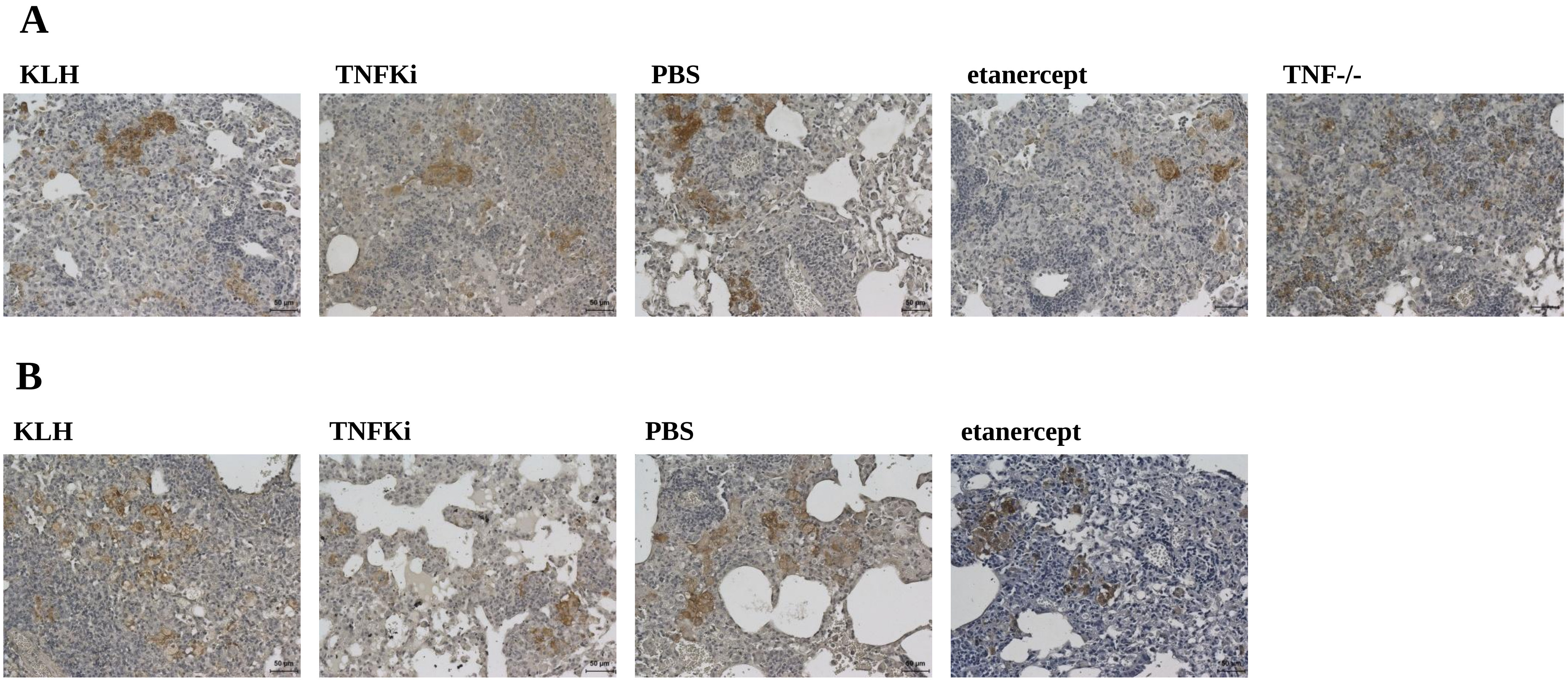

Supplementary figure 4: TNFKi vaccination does not inhibits iNOS expression in lungs of *M. tuberculosis* infected mice. Immunohistological staining of iNOS was performed as described in materials and methods. At early phase of infection (**A**), TNFKi, KLH and PBS groups were characterized by iNOS+ macrophage infiltration of lesions and very low number of unstained neutrophils. In $TNF^{-/-}$ and etanercept groups, iNOS was only expressed by macrophages, closed to altered neutrophils accumulations and necrosis area. At established phase of infection (**B**), etanercept group, presented more macrophages infiltration and foamy histiocytes expressing iNOS and less neutrophils than in early phase of infection. Lesions of PBS group presented very few neutrophils, and iNOS was expressed in macrophages and foamy histiocytes. Vaccinated mice with KLH or TNFKi presented similar cellular infiltration than PBS group excepted a lower number of foamy histiocytes. Scale bar, 200µm.

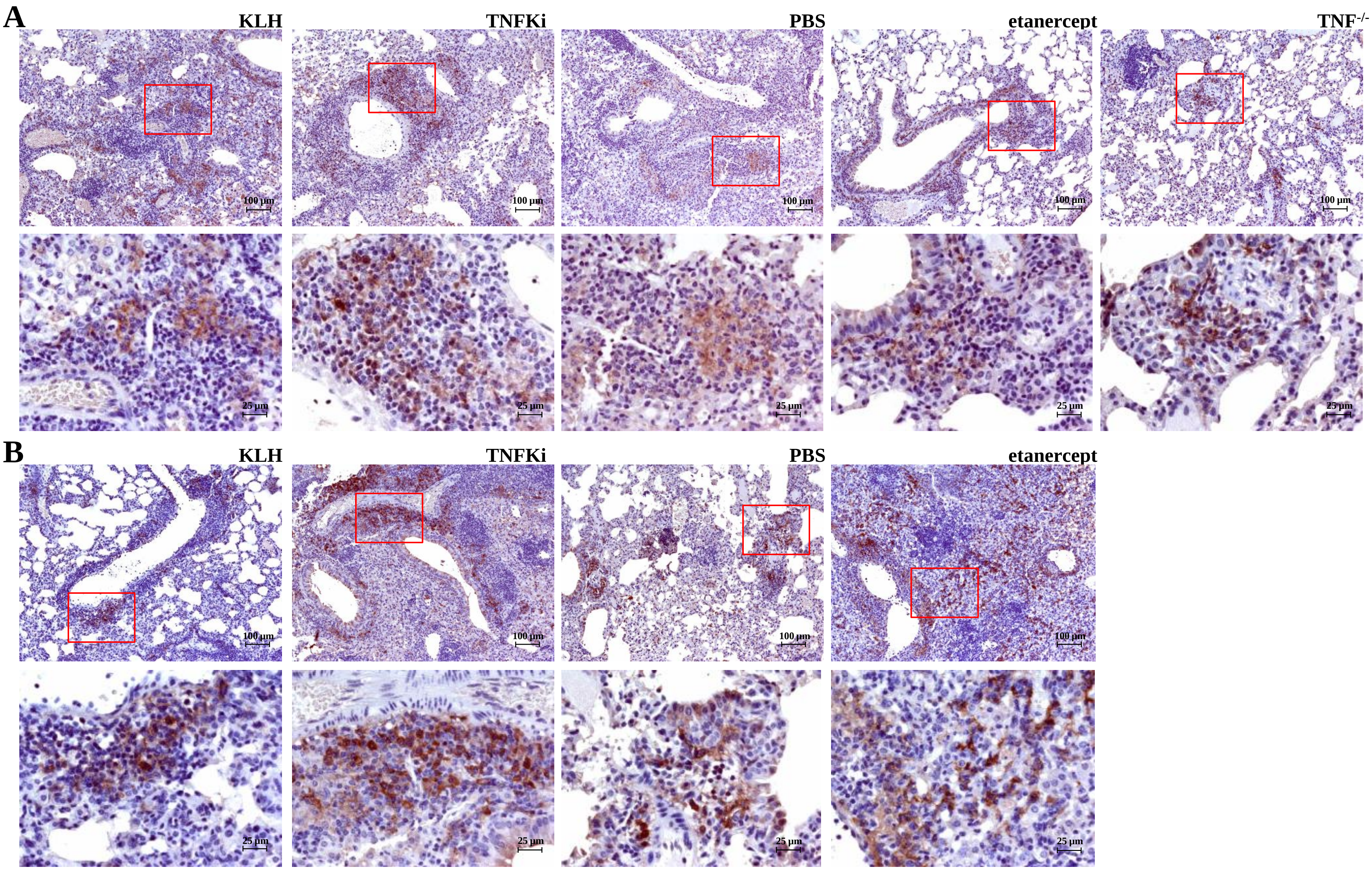

Supplementary figure 5: TNFKi vaccination does not inhibits macrophage infiltration in lungs of *M. tuberculosis* infected mice. Immunohistological staining of macrophages was performed using anti-F4/80 (clone BM8) antibodies. At early phase of infection (**A**), TNFKi, KLH and PBS groups presented similar macrophage infiltration of lesions and lung tissue. In $TNF^{-/-}$ and etanercept groups, macrophages were localized in lesions, closed to necrosis area. At established phase of infection (**B**), etanercept group, presented more macrophages infiltration than in early phase of infection. PBS group and mice vaccinated with KLH or TNFKi presented similar macrophage infiltration in both phases. Scale bar, 100 µm. X4 magnification of red boxes below original picture (Scale bar, 25 µm).

Treatments	Incidence of arthritis (n/group)	Onset of arthritis (days)	Maximal score (Amax /40)
TNFKi 20	5/6	38.7 +/- 2.5	8.0 +/- 2.2
TNFKi 10	5/6	38.2 +/- 2.4	4.7 +/- 2.4 *
TNFKi 5	6/6	38.8 +/- 1.6	8.0 +/- 1.4
KLH	6/6	33.3 +/- 1.7	14.7 +/- 2.1
PBS	6/6	33.3 +/- 2.2	11.1 +/- 3.3
etanercept	4/6	41.2 +/- 2.8	2.0 +/- 1.7 **

Supplementary table 1: TNFKi vaccination protects mice from Collagen-Induced Arthritis. DBA/1 mice were immunized at days -21, -7, 7 with KLH (n=6) or with different doses of TNFKi (20µg, 10µg, 5µg; n=6). Treatment with etanercept (30mg/kg, twice a week; n=6) or PBS (n=6) began from day 22 to day 47. Collagen-Induced Arthritis (CIA) were induced by two injections of CIIb (day 0 and 21). Three parameters of clinical arthritis were evaluated: incidence, onset and Amax. No statistical differences were obtained for incidence and onset of arthritis between treatment groups. By contrast maximal scores of arthritis (Amax) were lower in TNFKi and etanercept treated mice versus KLH group. * $p<0.05$ for TNFKi and ** $p<0.01$ for etanercept vs KLH (Newman-Keuls test with subsequent *post hoc* comparisons). CIA inhibition with TNFKi was observed in three independent experiments.
